# SARS-CoV-2 Vaccine Alpha and Delta Variant Breakthrough Infections Are Rare and Mild but Can Happen Relatively Early after Vaccination

**DOI:** 10.3390/microorganisms10050857

**Published:** 2022-04-21

**Authors:** Jelissa Katharina Peter, Fanny Wegner, Severin Gsponer, Fabrice Helfenstein, Tim Roloff, Rahel Tarnutzer, Kerstin Grosheintz, Moritz Back, Carla Schaubhut, Sabina Wagner, Helena M. B. Seth-Smith, Patrick Scotton, Maurice Redondo, Christiane Beckmann, Tanja Stadler, Andrea Salzmann, Henriette Kurth, Karoline Leuzinger, Stefano Bassetti, Roland Bingisser, Martin Siegemund, Maja Weisser, Manuel Battegay, Sarah Tschudin Sutter, Aitana Lebrand, Hans H. Hirsch, Simon Fuchs, Adrian Egli

**Affiliations:** 1 Department of Health Basel-City, 4052 Basel, Switzerland; jelissa.peter@hotmail.com (J.K.P.); severin.gsponer@bs.ch (S.G.); rahel.tarnutzer@bs.ch (R.T.); kerstin.grosheintz@bs.ch (K.G.); moritz.back@bs.ch (M.B.); carla.schaubhut@bs.ch (C.S.); sabina.wagner@bs.ch (S.W.); simon.fuchs@bs.ch (S.F.); 2 Applied Microbiology Research, Department of Biomedicine, University of Basel, 4056 Basel, Switzerland; fanny.wegner@usb.ch (F.W.); tim.roloff@usb.ch (T.R.); helena.seth-smith@usb.ch (H.M.B.S.-S.); 3 Division of Clinical Bacteriology and Mycology, University Hospital Basel, 4031 Basel, Switzerland; 4 SIB Swiss Institute of Bioinformatics, 1015 Lausanne, Switzerland; aitana.lebrand@sib.swiss; 5 Department of Clinical Research, University Hospital Basel, 4031 Basel, Switzerland; fabrice.helfenstein@usb.ch; 6 Corona Vaccination Centre for the Canton of Basel-City, 4058 Basel, Switzerland; patrick.scotton@centramed.ch; 7 Viollier AG, 4123 Allschwil, Switzerland; maurice.redondo@viollier.ch (M.R.); christiane.beckmann@viollier.ch (C.B.); andrea.salzmann@viollier.ch (A.S.); henriette.kurth@viollier.ch (H.K.); hans.hirsch@usb.ch (H.H.H.); 8 Department of Biosystems Science and Engineering, ETH Zurich, 4058 Basel, Switzerland; tanja.stadler@bsse.ethz.ch; 9 Clinical Virology, University Hospital Basel, 4031 Basel, Switzerland; karoline.leuzinger@usb.ch; 10 Transplantation & Clinical Virology, Department of Biomedicine, University of Basel, 4056 Basel, Switzerland; 11 Internal Medicine, University Hospital Basel, 4031 Basel, Switzerland; stefano.bassetti@usb.ch; 12 Emergency Medicine, University Hospital Basel, 4031 Basel, Switzerland; roland.bingisser@usb.ch; 13 Intensive Care Medicine, University Hospital Basel, 4031 Basel, Switzerland; martin.siegemund@usb.ch; 14 Infectious Diseases and Hospital Epidemiology, University Hospital Basel, University of Basel, 4031 Basel, Switzerland; maja.weisser@usb.ch (M.W.); manuel.battegay@usb.ch (M.B.); sarah.tschudin@usb.ch (S.T.S.)

**Keywords:** SARS-CoV-2, COVID-19, breakthrough infection, Alpha, Delta, COVID-19 vaccine, Moderna, Pfizer/BioNTech

## Abstract

(1) Background: Some COVID-19 vaccine recipients show breakthrough infection. It remains unknown, which factors contribute to risks and severe outcomes. Our aim was to identify risk factors for SCoV2 breakthrough infections in fully vaccinated individuals. (2) Methods: We conducted a retrospective case-control study from 28 December 2020 to 25 October 2021. Data of all patients with breakthrough infection was compared to data of all vaccine recipients in the Canton of Basel-City, Switzerland. Further, breakthrough infections by Alpha- and Delta-variants were compared. (3) Results: Only 0.39% (488/126,586) of all vaccine recipients suffered from a breakthrough infection during the observational period, whereof most cases were asymptomatic or mild (97.2%). Breakthrough infections after full vaccination occurred in the median after 78 days (IQR 47-123.5). Factors with lower odds for breakthrough infection were age (OR 0.987) and previous COVID-19 infection prior to vaccination (OR 0.296). Factors with higher odds for breakthrough infection included vaccination with Pfizer/BioNTech instead of Moderna (OR 1.459), chronic disease (OR 2.109), and healthcare workers (OR 1.404). (4) Conclusions: Breakthrough infections are rare and mild but can occur early after vaccination. This implies that booster vaccination might be initiated earlier, especially for risk groups. Due to new variants emerging repeatedly, continuous monitoring of breakthrough infections is crucial.

## 1. Introduction

The COVID-19 vaccines induce SARS-CoV-2 (SCoV2)-specific neutralizing antibodies [1,2,3,4] and reduce COVID-19-related hospitalization and mortality in randomized controlled clinical trials [5,6,7], as well as in the real world [8,9,10]. However, some vaccine recipients show breakthrough infection [5,11,12,13]. It is not fully understood which factors contribute to lower vaccine effectiveness [14,15] and how to best identify them for personalized vaccinations and booster strategies. Potential reasons include IgA deficiency, immunosuppressive drugs [16,17,18], or immunosenescence [19]. SCoV2-specific neutralizing antibodies slowly decrease over time and fall below a certain threshold, which results in a risk for breakthrough infection [20]. For this reason, additional vaccine boosters have become clinical practice in many countries by which neutralization titers can be elevated. The World Health Organisation recently stated that additional vaccination at standard doses or boosters at reduced doses should be firmly evidence-driven and targeted to the population groups in greatest need [21].

Viral evolution also contributes to risks of breakthrough infections, as observed for influenza [22]. Emerging new SCoV2 variants may reduce vaccine effectiveness by modulating the viral spike (S)-protein in the receptor binding domain (RBD) [23,24], which is critical for binding to the host’s angiotensin converting enzyme (ACE)-2 receptor [25,26,27]. Certain mutations in the viral S-protein have been retained and spread globally, whereby the Delta variant (B.1.617.2) has been rapidly displacing most of the other circulating variants. The efficiency to neutralize the Delta variant is lower compared to wild type or Alpha (B.1.1.7) variants. Two-times vaccinated individuals showed a 2.5-fold (Pfizer/BioNTech, Moderna and Janssen vaccine) reduction in neutralization titers against Delta compared to an ancestral G614 S pseudo-virus [28] and a 3-fold (Pfizer/BioNTech) and 6-fold (AstraZeneca) reduction in neutralization titers against Alpha [24]. Rapid molecular epidemiological adaptations and introductions of Delta and Omicron variants makes it more difficult to conclusively define such risk factors [29,30]. The new Omicron variant may show a 40-fold reduction to be neutralized in double dose vaccinated people [31].

Currently, there are two questions for public health decision making. Firstly, what are the possible demographic and clinical risk factors for SCoV2 breakthrough infections in vaccinated individuals? Knowledge would allow us to make general recommendations and prioritize third dose applications. Secondly, do patient characteristics differ in breakthrough infections caused by Alpha or Delta? The answer could help to link viral- and host-factors and learn for future emerging variants, including the currently dominating Omicron variant. 

Since the beginning of the Swiss COVID-19 vaccination campaign in December 2020, we have documented all breakthrough infections of the Canton of Basel-City. We conducted an exploratory retrospective case-control study and aimed to identify factors potentially associated with breakthrough infections in fully vaccinated people. We use an anonymized dataset of more than 120,000 fully vaccinated residents. We then investigated potential differences in breakthrough infections with either the Alpha or the Delta variants.

## 2. Materials and Methods

### 2.1. Ethics

The study has been approved by the local ethical committee (EKNZ 2020-00769 and 2021-00774). The data protection office of the Canton of Basel-City also evaluated and approved the study. 

### 2.2. Setting

The Canton of Basel-City has three municipalities with a total population of 201,386 people (June 2021) and is the most densely populated Swiss canton (5433/km^2^) with a population density comparable to Madrid (5481/km^2^), London (5701/km^2^), and Boston (5397/km^2^) [32,33,34,35]. The City of Basel itself is inhabited by 86% of the canton’s population. The first COVID-19 vaccine in the Canton of Basel-City was administered on the 28 December 2020, only 20 days after the launch of the global vaccination program. In the supplementary material, a detailed description of the time course on vaccination is available (Supplementary Material). By the 25 October 2021 126,586 individuals, equalling 63% of the canton’s population (201,354 total inhabitants in October 2021 [36]), were fully vaccinated.

### 2.3. Study Design and Population 

We conducted an exploratory retrospective case-control study from 28 December 2020 to 25 October 2021. Inclusion criteria for the case cohort were: (i) Individuals fully vaccinated in the Canton of Basel-City, including in retirement and nursing homes and hospitals, (ii) people living in the Canton of Basel-City: including Basel, Bettingen, and Riehen, and (iii) people with either SCoV2 specific NAT or antigen-test-confirmed infection. Exclusion criteria for the case cohort was a positive NAT result considered as residual finding from a SARS-CoV-2 infection before the final vaccination and individuals with breakthrough infection vaccinated with Janssen (n = 3) or AstraZeneca (n = 1). Inclusion criteria for the control cohort were: (i) Individuals fully vaccinated at the cantonal vaccination center or at health care institutions, pharmacies, and hospitals; and (ii) people living in the Canton of Basel-City including Basel, Bettingen, and Riehen. 

### 2.4. Data Collection

Data on all vaccinated individuals were collected from the Vaccination Monitoring Data Lake (VMDL) of Switzerland and the Corona Vaccination Centre for the Canton of Basel-City. All vaccinating sites in Switzerland are obliged to report to the national register according to the Swiss Epidemic law, with a 100% coverage of the canton’s total population. Data on positive tested individuals with a breakthrough infection were collected through the cantonal contact tracing database. All individuals with residence in the Canton of Basel-City tested positive for SCoV2 (RNA and antigen, self-tests not included) are being reported to the Federal Office of Public Health (FOPH) and through there to the cantonal Contact Tracing. Thereafter, each positive tested individual is contacted and monitored throughout the isolation, whereby personal and clinical information is being collected for source identification. For hospitalized individuals, the clinical information is being collected through the attending physician or nurse. We identified two sub-control groups: control group 1 comprises individuals fully vaccinated against COVID-19, who got vaccinated in all health care institutions, pharmacies, and hospitals, including the vaccine center in Basel City. Control group 2 is a subgroup of control group 1 and comprises individuals fully vaccinated against COVID-19 in the Corona Vaccination Centre for the Canton of Basel-City. Although meant as a control set, both control groups also comprise individuals who suffered from a breakthrough infection. Anonymization prevents tracking back individual information and thus prevents the exclusion of individuals with a breakthrough infection from the control sets.

Vaccine recipient characteristics have been collected as self-reported variables and were used to describe cases, to explain the probability of undergoing a breakthrough infection, or to explain the probability of undergoing a breakthrough infection caused by either the Alpha or Delta SCoV2 variants. Supplementary Table S1 shows an overview on control groups and patients with breakthrough infections (Supplementary Table S1).

The clinical course of infection was classified according to the National Institute of Health (NIH) COVID-19 treatment guidelines into “asymptomatic infection” (no symptoms), “mild illness” (various signs e.g., fever, cough; but no shortness of breath, dyspnoea or abnormal chest imaging), “moderate illness” (evidence of lower respiratory disease and oxygen saturation (SpO2) ≥ 94% on room air at sea level), “severe illness” (SpO2 < 94% on room air at sea level, PaO2/FiO2 < 300 mm Hg, respiratory frequency <30 breaths/min, or lung infiltrates >50%), “critical illness” (respiratory failure, septic shock, and/or multiple organ dysfunction) and “death” [37].

### 2.5. SCoV2 Lineage Determination

At the University Hospital Basel, we used viral whole genome sequencing to determine the Pango lineage in cases identified as breakthrough infection by Health Services of the Canton of Basel-City. Briefly, we used the Artic primers and sequenced amplicons on a NextSeq 500 platform (Illumina, San Diego, CA, USA), followed by the COVGAP pipeline to determine the viral lineage as previously described [38,39]. A subset of samples was sequenced in another laboratory. All sequences passing QC can be accessed via the Swiss Pathogen Surveillance Platform (www.spsp.ch, (accessed on 12 November 2021)) and ENA and GISAID accessions. The accession numbers of the genomes used for this analysis are available in the Supplementary Table S2.

### 2.6. Full Vaccination

Full vaccination was defined as (a) seven days after the second BNT162b2 vaccine (Pfizer/BioNTech) or (b) seven days after the first BNT162b2 vaccine when the patient had a confirmed SCoV2 infection at least 30 days before the dose, (c) 14 days after the second mRNA-1273 (Moderna) vaccine or (d) 14 days after the first mRNA-1273 when the patient had a confirmed SARS-CoV-2 infection at least 30 days before the single dose. The amount of buffer days complies with the expected time from vaccination to full protection defined in the respective clinical trials [1,3].

### 2.7. Breakthrough Infection

A breakthrough infection was defined by a laboratory-confirmed SCoV2 infection through either SCoV2-specific nucleic acid testing (NAT) or antigen testing at a health care center (antigen self-tests excluded) in fully vaccinated individuals. In Switzerland, positive antigen tests are recommended to be confirmed by NAT to exclude the rare possibility of false-positive results whenever possible, thereby also providing access to samples for SCoV2 genome sequencing. The date of the breakthrough infection was set to the date of sample collection. 

## 3. Results

### 3.1. Occurrence of Breakthrough Infections

**Figure 1:**
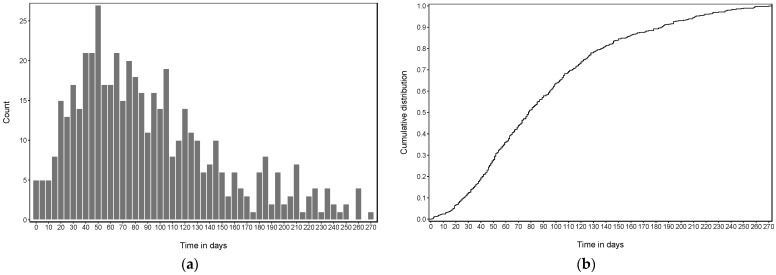
Distribution time in days from full vaccination (as defined above) to the positive SCoV2 test result confirming all cases of breakthrough infection. (**a**) Absolute frequency; (**b**) cumulative frequency.

We identified a total of 488 patients (0.39%) with breakthrough infections in an overall population of 126,586 vaccine recipients. First, we analyzed the time distribution, in days, elapsed from full vaccination to the positive SCoV2 test result for all patients with breakthrough infections. The time to a positive SCoV2 test shows that most breakthrough infections occurred between a few days to about 170 days after full vaccination, with a median of 78 days (interquartile range, IQR 47–123.5 days). The data suggests a weak bi-modal distribution, with a much weaker peak after 180 days. Accordingly, a steady increase of breakthrough infections starts after about 10–20 days post-vaccine and gradually levels off after about 130 days in the frequency and cumulative frequency distributions (Figure 1a,b). The burden of breakthrough infections correlated with the occurrence of overall SCoV2 cases in the observed community (Supplementary Figure S1). 

### 3.2. Clinical Case Presentation

We further analyzed all available patient characteristics for vaccine recipients, who suffered from a breakthrough infection (Supplementary Table S3). Briefly, patients with a breakthrough infection had a median age of 44.5 years (IQR 32-64) and were slightly more often female (52.7%). The mRNA-1273 (Moderna (Cambridge, MA, USA)) and BNT162b2 mRNA (Pfizer (New York, NY, USA)/BioNTech (Mainz, Germany)) were the two most commonly used COVID-19 vaccines in our region, accounting for 99.2% of vaccine applications. Generally, people with breakthrough infections had an asymptomatic presentation (26/488, 5.3%) or mild illness (448/488, 91.8%). Only a minority of patients required hospitalization during COVID-19 infection. However, it is unknown what percentage was hospitalized due to the disease (14/488, 2.9%). Severe illness (5/488, 1%) and critical illness or death (2/488, 0.4%) were very rare. Patients who showed moderate, severe, and critical illness also showed higher age (77, 81, and 87 years) and were having more predisposing risk factors compared to patients with asymptomatic or mild disease, e.g., cardiac disease 71.4, 80, and 100% (Supplementary Table S4). In a subset of patients, we could also determine the Alpha (n = 12) and Delta (n = 256) SCoV2 variants. The Delta variant showed more breakthrough infections compared to the Alpha variant in absolute numbers. Of note, this reflected the epidemiological situation in the general population during the observational period, where the Delta variant was the dominant virus lineage in Fall 2021.

Next, we compared patients with breakthrough infections to all vaccine recipients using the two control groups: control group 1 (n = 126,586) and 2 (n = 109,382) (Table 1). The median age in patients with SCoV2 breakthrough infection was slightly lower compared to the control group 1 and 2 (median 44.5 years vs. 49 years vs. 52 years). The patients with SCoV2 breakthrough infections were more frequently vaccinated with the Pfizer/BioNTech vaccine compared to the control group 1 and 2 (47.3 vs. 33.5 vs. 33.1%). Also notable is that people at especially high risk according to the Federal Office of Public Health, e.g., with a chronic disease (detailed list of conditions in Supplementary Table S5, which is a key indication for vaccination) suffered more frequently from breakthrough infection than the control group 1 and 2 (32 vs. 20.5 vs. 22.4%). Next, we compared characteristics, which were only available in patients with a breakthrough infection and people of control group 2 (which is a subset of control group 1). We observed slightly more breakthrough infections in patients with severe immunosuppression (5.1 vs. 3%) (Table 1). 

### 3.3. Identification of Potential Risk Factors

Next, we identified potential risk factors related to the probability of suffering from a breakthrough infection using generalized linear models. We compared patients with a breakthrough infection towards control group 1 (Table 2) and control group 2 (Supplementary Table S6). Overall, both multivariate regression models including control group 1 and 2 showed comparable results. 

**Figure 2:**
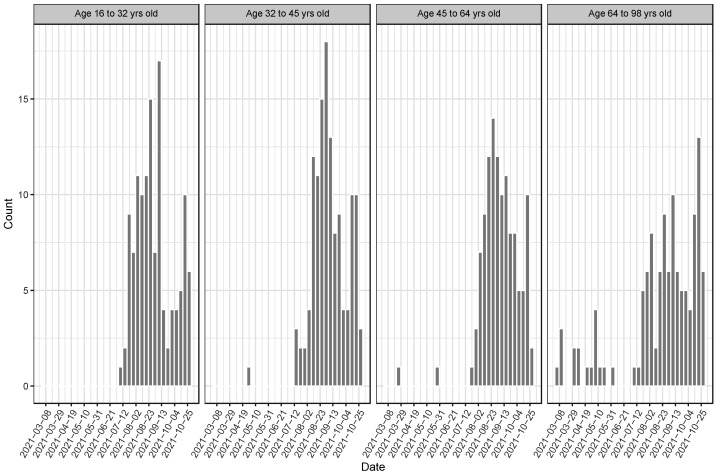
Distribution of dates of positive test for all patients with a breakthrough infection and according to age quartiles. In older patients (64 to 98 years old) first bulk of positive tests (from 1 March to 28 June 2021) is due to breakthrough infections caused by the Alpha variant.

A higher age was associated with lower odds for breakthrough infections (OR 0.987, 95% CI 0.983–0.992). Therefore, we further explored whether breakthrough infections were differently distributed between the age quartiles. Whereas 16 to 64 years old patients showed breakthrough infections mainly from July to October 2021, corresponding to infections caused by the Delta SCoV2 variant, patients above 64 years old showed a first bulk of breakthrough infections from January to May 2021, corresponding to infections caused by the Alpha variant, and a second, more important bulk from July to October corresponding to infections caused by the Delta variant (Figure 2), suggesting a different epidemiology as younger age groups could not get the vaccine as early, hence no breakthrough infection was possible. 

Breakthrough infections were more common in people vaccinated with the Pfizer/BioNTech vaccine (OR 1.459), with chronic disease (OR 2.109) and in healthcare workers (OR 1.404). Patients who recovered from COVID-19 prior to vaccination were less likely to suffer from a breakthrough infection (OR 0.296). We did not observe a significantly increased association for immunosuppressed patients (OR 1.248, 95% CI 0.806–1.849) (Supplementary Table S7). We also performed a subgroup analysis focusing on breakthrough infection with the Delta variant (Supplementary Table S8).

### 3.4. Difference between Breakthrough Infections with Alpha or Delta ScoV2 Variants

Next, we explored potential differences between breakthrough infections caused by the Alpha or Delta SCoV2 variants (Table 3). Supplementary Figures S2 and S3 show detailed time distributions for breakthrough infection caused by these variants (Supplementary Figures S2 and S3). Supplementary Figure S4 shows the probability of being free from breakthrough infection in relation to time for the two respective variants (Supplementary Figure S4). Based on the Swiss national surveillance program for SCoV2 and shared data efforts of the Swiss Pathogen Surveillance Platform (www.spsp.ch, (accessed on 2 December 2021)), we know that during Fall 2021 about 97% of the circulating viruses in Switzerland were Delta variants and subtypes thereof (https://www.covid19.admin.ch/en/, (accessed on 25 November 2021)). The sequences of the viral isolates identified are part of normal phylogenetic distribution and do not show specific unusual mutations, e.g., in the viral Spike protein (data not shown). During the observational period we did not observe the Omicron variant (B.1.529).

The Alpha variant was dominating from February to June 2021 and then replaced by the Delta variant in May 2021. During this time also, the vaccination concept and availability changed by providing access to individuals without high-risk disease and younger people. No difference regarding the time to positive test could be observed between the two variants. However, the two variants showed very different baseline characteristics. The median age for patients with breakthrough infection caused by the Alpha variant was 82 years and for the Delta variant only 43.5 years. This shows that breakthrough infections over time occurred in very different populations. The age difference is also reflected in the distribution of reasons for vaccination, e.g., the proportion of chronic diseases (91.7% in Alpha vs. 29.3% in Delta). Although the median time, in days, from considered fully vaccinated to a confirmed SCoV2 breakthrough infection was different with 42 days in the Alpha variant compared to 77 days in the Delta variant, this difference was not significant. It also seems that case severity in Alpha variants was higher compared to Delta variants, e.g., severe and critical illnesses accounted for 25% (Alpha) and 1.2% (Delta), which could also reflect the same issue of a more vulnerable population. For the Alpha variant, breakthrough infection was also more often linked to retirement and nursing homes (41.7%), whereas for the Delta variant it was mainly linked to holidays and work (24.6 and 6.6%).

Supplementary Table S8 summarizes the results of a generalized linear model with binomial distribution investigating potential risk factors associated with the relative probability of being infected by the Delta variant (vs. Alpha variant taken as a reference) in case of breakthrough infection (Supplementary Table S8). 

## 4. Discussion

Our study explores the key factors associated with COVID-19 vaccine breakthrough infection and compares the patient characteristics between people with a breakthrough infection caused by either the Alpha or Delta SCoV2 variant. The key findings of our study are: (i) breakthrough infections are very rare with only 0.39% (488/126,586) of the vaccinated population being affected during a 10-month observation period; (ii) breakthrough infections were either asymptomatic or mild (97.2%); (iii) most of the breakthrough infections in our population occurred between a few days to about 170 days after full vaccination, with a median of only 78 days for the time from being considered fully vaccinated (7 days (Pfizer/BioNTech) or 14 days (Moderna) after second vaccine or single vaccine following previous infection) to a positive SCoV2 test. We found no evidence that the protection offered by the vaccine fades away after some time. Further, the time elapsed from vaccination to breakthrough infection did not differ between the Alpha and the Delta variant.

In our cohort, older individuals are less likely to suffer from a breakthrough infection. Although elderly individuals are expected to be at higher risk due to immunosenescence, this finding might be explained by younger individuals being more likely to travel, enjoy social events outside their home, and thus be more exposed to the Delta variant, which caused most of the breakthrough infections in our study [40,41]. Especially after vaccination, young and healthy individuals might feel secure, leading to a higher risk behaviour. Similarly, individuals with close contact to a high-risk person were less likely to suffer from a breakthrough infection. This finding is most probably explained due to the more vigilant behaviour as well and underlines the fact that incautious behaviour might be a greater risk factor for breakthrough infection than intrinsic risk factors such as age or chronic disease. 

We also observed that individuals who recovered from a previous COVID-19 infection are less likely to suffer from a breakthrough infection. This is of interest particularly because the previous infection was caused by the Alpha variant whereas the breakthrough infection was caused by the Delta SCoV2 variant in this patient subgroup. The natural infection may build different types of immune response and additional immunological memory against other viral proteins [42,43] recently termed hybrid immunity [44]. Further studies regarding breakthrough infections in COVID-19 recovered patients will show whether a previous infection with the Alpha or Delta SARS-CoV-2 variant leads to sufficient protection against the dominating Omicron virus variant [45,46].

Our study indicates that individuals with a chronic disease are more likely to suffer from a breakthrough infection. Similar findings have been made mainly for the elderly with overall more chronic diseases [14,47,48]. 

Individuals vaccinated with the Pfizer/BioNTech vaccine (BNT162b2) are more likely to suffer from a breakthrough infection than individuals vaccinated with the Moderna vaccine (mRNA-1273). Tenforde and colleagues also showed that the mRNA-1273 vaccine was significantly more protective (OR, 0.11) compared with the BNT162b2 vaccine (OR, 0.19). The protective association against hospitalization for the BNT162b2 vaccine more than 120 days following vaccination declined notably (OR, 0.36; median, 143 days from vaccine dose 2 to illness onset), whereas the effectiveness of the mRNA-1273 vaccine more than 120 days post-vaccination was largely preserved (OR, 0.15; median, 141 days from vaccine dose 2 to illness onset) 6. Our study provides important confirmation and points out that risk groups for more severe clinical outcomes may benefit from the Moderna vaccine, due to the reduction in breakthrough infections.

Of particular relevance for future emerging variants and the current Omicron wave is our observation that healthcare workers are more likely to suffer from a breakthrough infection. This is an important finding and highlights the importance for timely booster vaccines in this cohort. A publication showed 39 cases of breakthrough infection in a total of 1497 healthcare workers, which was particularly linked to titers of SARS-CoV-2 neutralizing antibodies 5 and another study identified 4 of 1388 (0.3%) breakthrough infections in fully vaccinated compared to 21 of 585 (3.6%) in non-vaccinated staff of a long-term care facility. Most likely, the increased risk of breakthrough infection in healthcare workers is explained through a higher exposure to infected people. 

Further, in our cohort, most breakthrough infections were caused by the Delta variant. However, interpreting the temporal dynamics of infection relative to vaccination for the Alpha and Delta variant is difficult since these variants emerged at different time points relative to the availability of vaccination in Switzerland. Our additional models, focussing only on individuals vaccinated after the emergence of the Delta variant, show a median time of 50 days (IQR 30-72) to SCoV2-positive testing. As the subgroup analysis resulted in a loss of statistical power, the cumulative distributions no longer can resolve whether or not a breakthrough infection is more likely to occur as more time elapsed since full vaccination. Other studies have reported an increasing risk with a longer time since vaccination [20]. In our vaccine control cohort, the median time since vaccination was 115 days (IQR 84–145, min = 0, max = 273) and for individuals vaccinated after the Delta outbreak 82 days (IQR 51–100, min = 0, max = 111). The hypothesis of breakthrough infections increasing with elapsed time since full vaccination must consider new circulating SCoV2 variants and the overall increase in case numbers during this period. Indeed, our results are consistent with the notion that individuals infected with the Delta variant were at higher risk for breakthrough infection compared to individuals infected with the Alpha variant. Although, we have not determined prospectively neutralization antibody titers, this phenomenon is most likely explained by the amino acid changes on the virus spike protein and subsequently the resulting lower neutralization efficiency [24,28] and the general higher number of infected individuals with the Delta variant.

Surveillance of viral evolution using whole genome sequencing on a national scale remains critical for the months to come. As shown in our data as well, the clinical course of vaccine breakthrough infection has been described as generally mild (https://www.cdc.gov/coronavirus/2019-ncov/vaccines/effectiveness/why-measure-effectiveness/breakthrough-cases.html, (accessed on 20 October 2021)), with a few exceptions [49]. It is therefore crucial to also closely monitor the frequency and severity of SARS-CoV-2 breakthrough infections, including the clinical course, and combine this information with surveillance of viral evolution associated with reducing vaccine effectiveness, and their clinical course in fully vaccinated individuals with breakthrough infection. 

### Limitations

Our study has several important limitations. During the study timeframe, diagnostic and screening tests on SARS-CoV-2 for vaccinated individuals were only recommended by the Federal Office of Public Health (FOPH) when symptoms occurred or when the vaccinated individual lived in the same household as an individual tested positive for SARS-CoV-2. Therefore, although all positive test results are being reported to the FOPH, missing data on breakthrough infections in, e.g., asymptomatic patients is possible. Due to the fact that the study relies on real-world evidence data, a selection and reporting bias cannot be excluded. We included a relatively small number of sequenced cases with breakthrough infection—sequencing was available only for a subset of PCR confirmed cases (approximately 5–10%) of all cases are part of a nationwide surveillance program. Although we have not sequenced all strains, it is unlikely that we have missed a substantial dominant new lineage causing breakthrough infection, as the lineages dominating during the observational period were also found in other parts of the country as dominant lineages and also in the surrounding neighboring countries. Additionally, we do not have neutralizing antibody titers available for the whole cohort, however, this is also not a suitable assay for a general and large population. Interpreting the time of infection relative to vaccination is difficult due to the real-world study setting. The results might be confounded by pharmaceutical and non-pharmaceutical public health measures during the course of the study, incidence over time, population behaviour, and the temporal dynamics of the virus itself, with the Alpha variant being responsible for all the early breakthrough infections (before 6 July 2021) when mostly elderly people and people with comorbidities were getting vaccinated, and the Delta variant being responsible for a bulk of late breakthrough infections (after 6 July 2021). 

## 5. Conclusions

Overall, our study shows that breakthrough infections are rare and mild, and that even early after vaccination there might be a high risk for infection. In our study population, more than 50% of breakthrough infections occurred 70 to 80 days post-vaccine. This implies that possibly booster vaccination should be initiated earlier. Especially for risk groups associated with more frequent breakthrough infections such as healthcare workers and people in high-risk care facilities, this might be an important finding. Due to changes in the epidemiological dynamic with new variants emerging constantly, continuous monitoring of breakthrough infection will be very helpful to provide evidence on booster vaccines and patient groups at risk for potential complications.

## Figures and Tables

**Table 1:** Multivariable Comparison of patients with breakthrough infections compared to all control vaccine recipients using the control sets. Categorical variables are presented with numbers and percentages. * Serious side effects in previous vaccines focuses on any vaccine and is a self-reported variable. Since age shows some departure from normality in the case group, it is presented with median and [interquartile range].

	Level	Breakthrough	Control 1	Control 2	% Missing
n		488	126,586	109,382	
Gender (%)	Man	231 (47.3%)	60,640 (47.9%)	52,768 (48.2%)	0
	Woman	257 (52.7%)	65,944 (52.1%)	56,610 (51.8%)	
Age, median (IQR)		44.5 (32–64)	49 (34–65)	52 (36–68)	0
Type of vaccine, n (%)	Moderna	257 (52.7%)	84,200 (66.5%)	73,125 (66.9%)	0
	Pfizer/BioNTech	231 (47.3%)	42,386 (33.5%)	36,257 (33.1%)	
Indication for vaccination					
Chronic disease, n (%)	No	332 (68%)	100,668 (79.5%)	84,756 (77.6%)	0.1
	Yes	156 (32%)	25,918 (20.5%)	24,526 (22.4%)	
Healthcare worker, n (%)	No	429 (87.9%)	114,221 (90.2%)	101,868 (93.2%)	0.1
	Yes	59 (12.1%)	12,365 (9.8%)	7414 (6.8%)	
Close contact to high-risk person, n (%)	No	363 (74.4%)	94,629 (74.8%)	82,007 (75.0%)	0.1
	Yes	125 (25.6%)	31,957 (25.2%)	27,275 (25.0%)	
Serious side-effects previous vaccine *, n (%)	No	487 (99.8%)	N/A	108,194 (99.0%)	0.1
	Yes	1 (0.2%)		1084 (1.0%)	
Severe immunosuppression, n (%)	No	463 (94.9%)	N/A	105,994 (97.0%)	0.1
	Yes	25 (5.1%)		3288 (3.0%)	
Acute fever during time of vaccination n, (%)	No	488 (100%)	N/A	106,505 (100%)	2.6
	Yes	0 (0%)		46 (0%)	
Pregnant (%)	No	487 (99.8%)	124,482 (99.7%)	107,790 (99.8%)	1.3
	Yes	1 (0.2%)	339 (0.3%)	215 (0.2%)	
Recovered from COVID-19 prior to vaccination (%)	No	482 (98.8%)	121,730 (96.2%)	106,178 (97.1%)	0
	Yes	6 (1.2%)	4856 (3.8%)	3204 (2.9%)	

**Table 2:** Multivariable logistic regression exploring the relationships between potential risk factors and the probability to suffer from a SCoV2 breakthrough infection using the ‘control’ set 1. Only Moderna and Pfizer/BioNTech vaccines are compared because no other vaccine was used in the control population. A generalized linear mixed model was used.

	N	Odds Ratio	CI	*p*
Intercept	125,308	0.006	(0.005–0.008)	<0.001
Age		0.987	(0.983–0.992)	<0.001
Gender (woman vs. man)		1.037	(0.867–1.242)	0.692
Vaccine (Pfizer/BioNTech, ref = Moderna)		1.459	(1.238–1.612)	<0.001
Chronic disease (=Yes)		2.109	(1.692–2.620)	<0.001
Healthcare worker (=Yes)		1.404	(1.042–1.860)	0.022
Close contact with high-risk person (=Yes)		0.902	(0.724–1.116)	0.349
Pregnant (=Yes)		0.671	(0.038–2.995)	0.691
Recovered from COVID-19 prior to vaccination (=Yes)		0.296	(0.117–0.606)	0.003

**Table 3:** Comparisons of patient characteristics between individuals who suffered from a breakthrough infection caused by the Alpha or the Delta variant. Categorical variables are presented with numbers and percentages. * Serious side-effects in previous vaccines focuses on any vaccine and is a self-reported variable. Variables showing departure from normality are presented with median and [Interquartile Range].

Variable	Level	SCoV2-Alpha	SCoV2-Delta	% Missing
n		12	256	
Age, med (IQR)	Years	82.0 (70.5–87.0)	43.5 (32.0–61.8)	45.1
Sex, n (%)	Man	5 (41.7%)	123 (48%)	45.1
	Woman	7 (58.3%)	133 (52%)	
Type of vaccine, n (%)	Moderna	1 (8.3%)	136 (53.1%)	45.1
	Pfizer/BioNTech	11 (91.7%)	120 (46.9%)	
Indication for vaccination				
Chronic diseases, n (%)	No	1 (8,3%)	181 (70.7%)	45.1
	Yes	11 (91.7%)	75 (29.3%)	
Healthcare worker, n (%)	No	12 (100%)	221 (86.3%)	45.1
	Yes	0 (0%)	35 (13.7%)	
Close contact to high-risk person, n (%)	No	6 (50%)	190 (74.2%)	45.1
	Yes	6 (50%)	66 (25.8%)	
Serious side effects to other vaccines *, n (%)	No	12 (100%)	255 (99.6%)	45.1
	Yes	0 (0%)	1 (0.4%)	
Severe immunosuppression, n (%)	No	9 (75%)	245 (95.7%)	45.1
	Yes	3 (25%)	11 (4.3%)	
Acute fever, n (%)	No	12 (100%)	256 (100%)	45.1
	Yes	0 (0%)	0 (0%)	
Pregnant, n (%)	No	12 (100%)	255 (99.6%)	45.1
	Yes	0 (0%)	1 (0.4%)	
Recovered from COVID-19 prior to vaccination, n (%)	No	12 (100%)	254 (99.2%)	45.1
	Yes	0 (0%)	2 (0.8%)	
Time vacc to 1st symptoms, median (IQR)	days	59 (57–61)	75 (44–118)	48.4
Time vacc/Delta outbreak to 1st symptoms, median (IQR)	days	59 (57–61)	50 (29–72.25)	49
Time vacc to 1st symptoms for Alpha & Delta patients vaccinated after Delta outbreak, median (IQR)	days	59 (57–61)	40 (23–57)	48
Time vacc to positive test, median (IQR)	days	42 (17–63)	77 (47–120)	45.3
Time vacc/Delta outbreak to positive test, median (IQR)	days	42 (17–63)	50 (32–72)	45.3
Time vacc to positive test for Alpha & Delta patients vaccinated after Delta outbreak, median (IQR)	days	42 (17–63)	45 (26–59)	82.2
PCR ct value, median (IQR)		20 (20–20)	19.99 (17.21–23.08)	70.9
Severity of disease, n (%)	Asymptomatic	2 (16.7%)	6 (2.3%)	45.1
	Mild illness	7 (58.3%)	242 (94.5%)	
	Moderate illness	0 (0%)	5 (2%)	
	Severe illness	2 (16.7%)	2 (0.8%)	
	Critical illness and death	1 (8.3%)	1 (0.4%)	
Hospitalisation	No	11 (91.7%)	148 (96.9%)	45.1
	Yes	1 (8.3%)	8 (3.1%)	
Suspected source of infection	Club, party, bar	0 (0%)	12 (4.7%)	45.1
	Cultural event	0 (0%)	4 (1.6%)	
	Family	3 (25%)	71 (27.7%)	
	Friends	0 (0%)	14 (5.5%)	
	Holidays	1 (8.3%)	63 (24.6%)	
	Hospital	1 (8.3%)	3 (1.2%)	
	Religion	0 (0%)	1 (0.4)	
	Retirement and nursing home	5 (41.7%)	10 (3.9%)	
	School	0 (0%)	1 (0.4%)	
	Sports	0 (0%)	3 (1.2%)	
	Unknown	2 (16.7%)	57 (22.3%)	
	Work	0 (0%)	17 (6.6%)	
Baseline risk factor				
Chronic lung disease	No	11 (91.7%)	241 (94.1%)	45.1
	Yes	1 (8.3%)	15 (5.9%)	
Chronic renal disease	No	11 (91.7%)	252 (98.4%)	45.1
	Yes	1 (8.3%)	4 (1.6%)	
Cancer	No	10 (83.3%)	251 (98%)	45.1
	Yes	2 (16.7%)	5 (2%)	
Cardiac disease	No	7 (58.3%)	238 (93%)	45.1
	Yes	5 (41.7%)	18 (7%)	
Arterial hypertension	No	9 (75%)	213 (83.2%)	45.1
	Yes	3(25%)	43 (16.8%)	
Diabetes	No	10 (83.3%)	232 (90.6%)	45.1
	Yes	2 (16.7%)	24 (9.4%)	
Obesity	No	11 (91.7%)	255 (99.6%)	45.1
	Yes	1 (8.3%)	1 (0.4%)	

## Data Availability

Not applicable.

## Supplementary Materials

The following supporting information can be downloaded at: https://www.mdpi.com/article/10.3390/microorganisms10050857/s1, Figure S1: SARS-CoV-2 PCR confirmed cases after introduction of the vaccine. The red line shows all breakthrough infections and the black line shows all PCR confirmed cases (secondary axis); Figure S2A,B: Distribution time in days from full vaccination (as defined) to the breakthrough infections with the Alpha variant; Figure S3A,B: Distribution time in days from full vaccination (as defined) to the breakthrough infections with the Delta variant; Figure S4: Kaplan–Meier curve representing the proportion of patients free of breakthrough infection in relation to time elapsed between the date of full vaccination and positive test for the Alpha and Delta variant; Table S1: Collected vaccine recipient and patient characteristics; Table S2: Accession numbers of used genomes in the analysis; Table S3: Baseline characteristics for all individuals in the database with confirmed breakthrough infection; Table S4: Baseline characteristics of patients with breakthrough infection who showed asymptomatic or mild illness compared to patients with moderate, severe, and critical illness; Table S5: Chronic diseases and conditions leading to an especially high risk for severe course of disease according to the FOPH, only adults are considered to be at high risk. Therefore, the criteria refer only to adults; Table S6: Multivariable logistic regression exploring the relationships between potential risk factors and the probability to suffer from a SARS-CoV-2 breakthrough infection using the ‘control’ group 2. Confidence intervals and p-values are computed using Firth penalization; Table S7: Multivariable logistic regression exploring the relationships between potential risk factors and the probability to suffer from a Delta variant caused breakthrough infection; Table S8: Multivariable generalized linear model with binomial distribution investigating the probability of suffering from a breakthrough infection caused by the Delta variant (as compared to the Alpha variant taken as the reference) as a function of individual characteristics. Confidence intervals and p-values are computed using Firth penalization due to rare or no observations in some factors’ levels; Material S1: Ethics, vaccine availability, statistical analysis.

## References

[1] Baden L.R., El Sahly H.M., Essink B., Kotloff K., Frey S., Novak R., Diemert D., Spector S.A., Rouphael N., Creech C.B. (2021). Efficacy and Safety of the mRNA-1273 SARS-CoV-2 Vaccine. N. Engl. J. Med..

[2] Dagan N., Barda N., Kepten E., Miron O., Perchik S., Katz M.A., Hernán M.A., Lipsitch M., Reis B., Balicer R.D. (2021). BNT162b2 mRNA COVID-19 Vaccine in a Nationwide Mass Vaccination Setting. N. Engl. J. Med..

[3] Polack F.P., Thomas S.J., Kitchin N., Absalon J., Gurtman A., Lockhart S., Perez J.L., Pérez Marc G., Moreira E.D., Zerbini C. (2020). Safety and efficacy of the BNT162b2 mRNA COVID-19 vaccine. N. Engl. J. Med..

[4] Stephenson K.E., Le Gars M., Sadoff J., de Groot A.M., Heerwegh D., Truyers C., Atyeo C., Loos C., Chandrashekar A., McMahan K. (2021). Immunogenicity of the Ad26.COV2.S Vaccine for COVID-19. JAMA J. Am. Med. Assoc..

[5] Bergwerk M., Gonen T., Lustig Y., Amit S., Lipsitch M., Cohen C., Mandelboim M., Levin E.G., Rubin C., Indenbaum V. (2021). COVID-19 Breakthrough Infections in Vaccinated Health Care Workers. N. Engl. J. Med..

[6] Tenforde M.W., Self W.H., Adams K., Gaglani M., Ginde A.A., McNeal T., Ghamande S., Douin D.J., Talbot H.K., Casey J.D. (2021). Association Between mRNA Vaccination and COVID-19 Hospitalization and Disease Severity. JAMA J. Am. Med. Assoc..

[7] Thompson M.G., Burgess J.L., Naleway A.L., Tyner H., Yoon S.K., Meece J., Olsho L.E.W., Caban-Martinez A.J., Fowlkes A.L., Lutrick K. (2021). Prevention and Attenuation of COVID-19 with the BNT162b2 and mRNA-1273 Vaccines. N. Engl. J. Med..

[8] Chemaitelly H., Tang P., Hasan M.R., AlMukdad S., Yassine H.M., Benslimane F.M., Al Khatib H.A., Coyle P., Ayoub H.H., Al Kanaani Z. (2021). Waning of BNT162b2 Vaccine Protection against SARS-CoV-2 Infection in Qatar. N. Engl. J. Med..

[9] Chodick G., Tene L., Rotem R.S., Patalon T., Gazit S., Ben-Tov A., Weil C., Goldshtein I., Twig G., Cohen D. (2021). The Effectiveness of the Two-Dose BNT162b2 Vaccine: Analysis of Real-World Data. Clin. Infect. Dis..

[10] Thompson M.G., Stenehjem E., Grannis S., Ball S.W., Naleway A.L., Ong T.C., DeSilva M.B., Natarajan K., Bozio C.H., Lewis N. (2021). Effectiveness of COVID-19 Vaccines in Ambulatory and Inpatient Care Settings. N. Engl. J. Med..

[11] Haas E.J., Angulo F.J., McLaughlin J.M., Anis E., Singer S.R., Khan F., Brooks N., Smaja M., Mircus G., Pan K. (2021). Impact and effectiveness of mRNA BNT162b2 vaccine against SARS-CoV-2 infections and COVID-19 cases, hospitalisations, and deaths following a nationwide vaccination campaign in Israel: An observational study using national surveillance data. Lancet.

[12] Hacisuleyman E., Hale C., Saito Y., Blachere N.E., Bergh M., Conlon E.G., Schaefer-Babajew D.J., DaSilva J., Muecksch F., Gaebler C. (2021). Vaccine Breakthrough Infections with SARS-CoV-2 Variants. N. Engl. J. Med..

[13] Thomas S.J., Moreira E.D., Kitchin N., Absalon J., Gurtman A., Lockhart S., Perez J.L., Pérez Marc G., Polack F.P., Zerbini C. (2021). Safety and Efficacy of the BNT162b2 mRNA COVID-19 Vaccine through 6 Months. N. Engl. J. Med..

[14] Lipsitch M., Krammer F., Regev-Yochay G., Lustig Y., Balicer R.D. (2021). SARS-CoV-2 breakthrough infections in vaccinated individuals: Measurement, causes and impact. Nat. Rev. Immunol..

[15] Liu C., Lee J., Ta C., Soroush A., Rogers J.R., Kim J.H., Natarajan K., Zucker J., Weng C. (2021). A Retrospective Analysis of COVID-19 mRNA Vaccine Breakthrough Infections—Risk Factors and Vaccine Effectiveness. medRxiv.

[16] Aslam S., Adler E., Mekeel K., Little S.J. (2021). Clinical effectiveness of COVID-19 vaccination in solid organ transplant recipients. Transpl. Infect. Dis..

[17] Brosh-Nissimov T., Orenbuch-Harroch E., Chowers M., Elbaz M., Nesher L., Stein M., Maor Y., Cohen R., Hussein K., Weinberger M. (2021). BNT162b2 vaccine breakthrough: Clinical characteristics of 152 fully vaccinated hospitalized COVID-19 patients in Israel. Clin. Microbiol. Infect..

[18] Qin C.X., Moore L.W., Anjan S., Rahamimov R., Sifri C.D., Ali N.M., Morales M.K., Tsapepas D.S., Basic-Jukic N., Miller R.A. (2021). Risk of Breakthrough SARS-CoV-2 Infections in Adult Transplant Recipients. Transplantation.

[19] Barda N., Dagan N., Cohen C., Hernán M.A., Lipsitch M., Kohane I.S., Reis B.Y., Balicer R.D. (2021). Effectiveness of a third dose of the BNT162b2 mRNA COVID-19 vaccine for pre-venting severe outcomes in Israel: An observational study. Lancet.

[20] Mizrahi B., Lotan R., Kalkstein N., Peretz A., Perez G., Ben-Tov A., Chodick G., Gazit S., Patalon T. (2021). Correlation of SARS-CoV-2-breakthrough infections to time-from-vaccine. Nat. Commun..

[21] World Health Organization (2021). Interim Statement on Booster Doses for COVID-19 Vaccination. https://www.who.int/news/item/04-10-2021-interim-statement-on-booster-doses-for-COVID-19-vaccination.

[22] Vilches T.N., Shoukat A., Ferreira C.P., Moghadas S.M. (2020). Projecting influenza vaccine effectiveness: A simulation study. PLoS ONE.

[23] Mlcochova P., Kemp S.A., Dhar M.S., Papa G., Meng B., Ferreira I.A.T.M., Datir R., Collier D.A., Albecka A., Singh S. (2021). SARS-CoV-2 B.1.617.2 Delta variant replication and immune evasion. Nature.

[24] Planas D., Veyer D., Baidaliuk A., Staropoli I., Guivel-Benhassine F., Rajah M.M., Planchais C., Porrot F., Robillard N., Puech J. (2021). Reduced sensitivity of SARS-CoV-2 variant Delta to antibody neutralization. Nature.

[25] Jackson C.B., Farzan M., Chen B., Choe H. (2022). Mechanisms of SARS-CoV-2 entry into cells. Nat. Rev. Mol. Cell Biol..

[26] Lan J., Ge J., Yu J., Shan S., Zhou H., Fan S., Zhang Q., Shi X., Wang Q., Zhang L. (2020). Structure of the SARS-CoV-2 spike receptor-binding domain bound to the ACE2 receptor. Nature.

[27] Li J., Lai S., Gao G.F., Shi W. (2021). The emergence, genomic diversity and global spread of SARS-CoV-2. Nature.

[28] McCallum M., Walls A.C., Sprouse K.R., Bowen J.E., Rosen L.E., Dang H.V., De Marco A., Franko N., Tilles S.W., Logue J. (2021). Molecular basis of immune evasion by the Delta and Kappa SARS-CoV-2 variants. Science.

[29] Puranik A., Lenehan P.J., Silvert E., Niesen M.J., Corchado-Garcia J., O’Horo J.C., Virk A., Swift M.D., Halamka J., Badley A.D. (2021). Comparison of two highly-effective mRNA vaccines for COVID-19 during periods of Alpha and Delta variant prevalence. medRxiv.

[30] Burki T.K. (2021). Omicron variant and booster COVID-19 vaccines. Lancet Respir. Med..

[31] Lu L., Mok B.W.Y., Chen L., Chan J.M.C., Tsang O.T.Y., Lam B.H.S., Chuang V.W.M., Chu A.W.H., Chan W.M., Ip J.D. (2021). Neutralization of SARS-CoV-2 Omicron variant by sera from BNT162b2 or Coronavac vaccine recipients. medRxiv.

[32] Swiss Federal Statistical Office (2021). Sustainable Development, Regional and International Disparities, Statistical Basis and Overviews.

[33] Bureau USC (2021). Boston City, Massachussetts. https://www.census.gov/quickfacts/fact/table/bostoncitymassachusetts/POP010220.

[34] Park N. (2021). Estimates of the Population for the UK, England and Wales, Scotland and Northern Ireland. https://www.ons.gov.uk/peoplepopulationandcommunity/populationandmigration/populationestimates/datasets/populationestimatesforukenglandandwalesscotlandandnorthernireland.

[35] (2021). Madrid Co. El Municipio en Cigfras. http://portalestadistico.com/municipioencifras/?pn=madrid&pc=ZTV21.

[36] Statistisches Amt des Kantons Basel-Stadt (2021). Bestand und Struktur. https://www.statistik.bs.ch/zahlen/tabellen/1-bevoelkerung/bestand-struktur.html.

[37] Bethesda (2021). Coronavirus Disease 2019 (COVID-19) Treatment Guidelines.

[38] Stange M., Mari A., Roloff T., Seth-Smith H.M., Schweitzer M., Brunner M., Leuzinger K., Søgaard K.K., Gensch A., Tschudin-Sutter S. (2021). SARS-CoV-2 outbreak in a tri-national urban area is dominated by a B.1 lineage variant linked to a mass gathering event. PLoS Pathog..

[39] Wegner F., Roloff T., Huber M., Cordey S., Ramette A., Gerth Y., Bertelli C., Stange M., Seth-Smith H.M., Mari A. (2021). External quality assessment of SARS-CoV-2-sequencing: An ESGMD-SSM pilot trial across 15 European laboratories. J. Clin. Microbiol..

[40] Antonelli M., Penfold R.S., Merino J., Sudre C.H., Molteni E., Berry S., Canas L.S., Graham M.S., Klaser K., Modat M. (2021). Risk factors and disease profile of post-vaccination SARS-CoV-2 infection in UK users of the COVID Symptom Study app: A prospective, community-based, nested, case-control study. Lancet Infect. Dis..

[41] Day M. (2021). COVID-19: Stronger warnings are needed to curb socialising after vaccination, say doctors and behavioural scientists. BMJ.

[42] Kojima N., Klausner J.D. (2021). Protective immunity after recovery from SARS-CoV-2 infection. Lancet Infect. Dis..

[43] Townsend J.P., Hassler H.B., Wang Z., Miura S., Singh J., Kumar S., Ruddle N.H., Galvani A.P., Dornburg A. (2021). The durability of immunity against reinfection by SARS-CoV-2: A comparative evolutionary study. Lancet Microbe.

[44] Crotty S. (2021). Hybrid immunity. Science.

[45] Wratil P.R., Stern M., Priller A., Willmann A., Almanzar G., Vogel E., Feuerherd M., Cheng C.C., Yazici S., Christa C. (2022). Three exposures to the spike protein Three exposures to the spike protein of SARS-CoV-2 by either infection or vaccination elicit superior neutralizing immunity to all variants of concern. Nat. Med..

[46] Lechmere T., Snell L.B., Graham C., Seow J., Shalim Z.A., Charalampous T., Alcolea-Medina A., Batra R., Nebbia G., Edgeworth J.D. (2022). Broad Neutralization of SARS-CoV-2 Variants, Including Omicron, following Breakthrough Infection with Delta in COVID-19-Vaccinated Individuals. Mbio.

[47] Klompas M. (2021). Understanding Breakthrough Infections Following mRNA SARS-CoV-2 Vaccination. JAMA.

[48] Stefan N. (2021). Metabolic disorders, COVID-19 and vaccine-breakthrough infections. Nat. Rev. Endocrinol..

[49] Wang S.Y., Juthani P.V., Borges K.A., Shallow M.K., Gupta A., Price C., Won C.H., Chun H.J. (2021). Severe breakthrough COVID-19 cases in the SARS-CoV-2 delta (B.1.617.2) variant era. Lancet Microbe.

