# Supplementary material for: SARS-CoV-2 Vaccine Alpha and Delta Variant Breakthrough Infections Are Rare and Mild but Can Happen Relatively Early after Vaccination
Source: Microorganisms. 2022 Apr 21;10(5):857. doi: 10.3390/microorganisms10050857 (PMC9146960; doi:10.3390/microorganisms10050857)
Supplement: Supplementary file 1 [file microorganisms-10-00857-s001.zip › microorganisms-1685866-supplementary.pdf]

microorganisms

MDPI

Supplementary materials

Table S1. Collected vaccine recipient and patient characteristics. * other reasons than age, healthcare worker, chronic disease, contact with vulnerable person, living in a community with increased risks. * general previous side effects.

Control group 1	Control group 2	Breakthrough infections
Age		Age
Gender, (male/female), count & percentage	Gender, (male/female), count & percentage	Gender, (male/female), count & percentage
Type of vaccine; categorical, Pfizer/BioNTech, Moderna	Type of vaccine; categorical, Pfizer/BioNTech, Moderna	Type of vaccine; categorical, Pfizer/BioNTech, Modern
Chronic disease, (Yes/No)	Chronic disease, (Yes/No)	Chronic disease, (Yes/No)
Healthcare worker, (Yes/No)	Healthcare worker, (Yes/No)	Healthcare worker, (Yes/No)
Close contact to high-risk person, (Yes/No)	Close contact to high-risk person, (Yes/No)	Close contact to high-risk person, (Yes/No)
Vaccination for other reasons*, (Yes/No)	N/A	
Pregnancy, (Yes/No)	Pregnancy, (Yes/No)	Pregnancy, (Yes/No)
Person recovered from COVID-19, (Yes/No)	Person recovered from COVID-19, (Yes/No)	Person recovered from COVID-19, (Yes/No)
Date of full vaccination	Date of full vaccination	Date of full vaccination
N/A	Severe immunosuppression, (Yes/No)	Severe immunosuppression, (Yes/No)
N/A	Serious previous side effects*, (Yes/No)	Serious side effects, (Yes/No)
N/A	Acute fever before / during COVID-19 vaccination, (Yes/No)	Acute fever before / during COVID-19 vaccination, (Yes/No)
N/A	N/A	Time between considered fully vaccinated and positive test
N/A	N/A	Time between considered fully vaccinated and first symptoms
N/A	N/A	SARS-CoV-2 variant, (Alpha or Delta), count & percent
N/A	N/A	Hospitalisation, (Yes/No)

N/A	N/A	Suspected source of infection: club, party, bar; cultural event; family; friends; holidays; hospital; religion; retirement and nursing home; school; sports; work; unknown;
N/A	N/A	Chronic lung disease, (Yes/No)
N/A	N/A	Chronic renal disease, (Yes/No)
N/A	N/A	Cancer, (Yes/No)
N/A	N/A	Cardiovascular disease, (Yes/No)
N/A	N/A	Arterial hypertension, (Yes/No)
N/A	N/A	Diabetes, (Yes/No)
N/A	N/A	Obesity, (Yes/No)

Table S2. Accession numbers of used genomes in this analysis.

GISAID Identifier	Pangolin Lineage
EPI_ISL_3086290	AY.4
EPI_ISL_4903597	AY.4
EPI_ISL_4903635	AY.102
EPI_ISL_5049804	AY.102
EPI_ISL_5307606	AY.46
EPI_ISL_5307608	AY.43
EPI_ISL_5307610	AY.54
EPI_ISL_5307620	AY.43
EPI_ISL_5307622	AY.14
EPI_ISL_5307667	AY.46
EPI_ISL_5307709	AY.102
EPI_ISL_5428846	AY.4
EPI_ISL_5428847	AY.41
EPI_ISL_5428848	AY.4
EPI_ISL_5428849	AY.46.6
EPI_ISL_5428850	AY.111
EPI_ISL_5508919	AY.43
EPI_ISL_5508977	AY.98.1

EPI_ISL_5508986	AY.98.1
EPI_ISL_5508994	AY.43
EPI_ISL_5509063	AY.80
EPI_ISL_5509099	AY.47
EPI_ISL_5509125	AY.33
EPI_ISL_5509131	AY.33
EPI_ISL_5509160	AY.43
EPI_ISL_5509166	AY.4
EPI_ISL_5765992	AY.43
EPI_ISL_5766012	AY.95
EPI_ISL_5766062	AY.43
EPI_ISL_5766070	AY.43
EPI_ISL_5766077	AY.43
EPI_ISL_5766144	AY.43
EPI_ISL_5766182	B.1.617.2
EPI_ISL_5766183	AY.20
EPI_ISL_5766195	B.1.617.2
EPI_ISL_5766203	AY.43
EPI_ISL_5766208	AY.43
EPI_ISL_5766269	AY.46.6
EPI_ISL_5841634	AY.44
EPI_ISL_5841635	AY.54
EPI_ISL_5841657	AY.4
EPI_ISL_5841661	AY.102
EPI_ISL_5841666	AY.43
EPI_ISL_5841685	AY.4
EPI_ISL_5841691	AY.102
EPI_ISL_5841709	AY.9.2
EPI_ISL_5933964	AY.102
EPI_ISL_5933970	AY.102
EPI_ISL_5934004	AY.4
EPI_ISL_5934005	AY.102

EPI_ISL_5934006	AY.43
EPI_ISL_5934007	AY.43
EPI_ISL_5934008	AY.4
EPI_ISL_5934009	AY.102
EPI_ISL_5934010	AY.46.6
EPI_ISL_6015872	AY.44
EPI_ISL_6015873	AY.43
EPI_ISL_6015874	AY.43
EPI_ISL_6015875	AY.43
EPI_ISL_6157569	AY.54
EPI_ISL_6157570	AY.102
EPI_ISL_3086299	AY.70
EPI_ISL_5841659	AY.43
EPI_ISL_1747622	B.1.1.7
EPI_ISL_1747636	B.1.1.7
EPI_ISL_1747641	B.1.1.7
EPI_ISL_1748263	B.1.1.7
EPI_ISL_1748463	B.1.1.7
EPI_ISL_1914607	B.1.1.7
EPI_ISL_2270095	B.1.1.7
EPI_ISL_2466583	B.1.1.7
EPI_ISL_3030104	AY.98.1
EPI_ISL_3030113	AY.43
EPI_ISL_3086318	AY.42
EPI_ISL_3086319	B.1.617.2
EPI_ISL_3184351	AY.43
EPI_ISL_3184366	AY.9.2
EPI_ISL_3229188	B.1.1.7
EPI_ISL_3229190	AY.43
EPI_ISL_3229201	AY.43
EPI_ISL_3229213	B.1.617.2
EPI_ISL_3229214	B.1.617.2

EPI_ISL_3229224	AY.5
EPI_ISL_3229225	AY.43
EPI_ISL_3229227	AY.43
EPI_ISL_3229248	AY.4
EPI_ISL_3247301	AY.43
EPI_ISL_3247308	AY.42
EPI_ISL_3247349	AY.98.1
EPI_ISL_3303743	AY.43
EPI_ISL_3303758	AY.42
EPI_ISL_3344075	AY.42
EPI_ISL_3344101	AY.4
EPI_ISL_3344102	AY.43
EPI_ISL_3344111	B.1.617.2
EPI_ISL_3425805	AY.98.1
EPI_ISL_3425806	AY.34
EPI_ISL_3451674	AY.43
EPI_ISL_3451732	AY.43
EPI_ISL_3451733	AY.43
EPI_ISL_3530135	AY.42
EPI_ISL_3530171	AY.94
EPI_ISL_3530179	AY.23
EPI_ISL_3530776	AY.43
EPI_ISL_3530792	AY.98.1
EPI_ISL_3530842	AY.34
EPI_ISL_3570114	AY.16
EPI_ISL_3570115	B.1.617.2
EPI_ISL_3570127	AY.43
EPI_ISL_3570128	B.1.617.2
EPI_ISL_3570130	AY.43
EPI_ISL_3710254	AY.4
EPI_ISL_3710255	AY.98.1
EPI_ISL_3730669	AZ.2

EPI_ISL_3730670	AY.36
EPI_ISL_3824224	AY.42
EPI_ISL_3824225	AY.4
EPI_ISL_3824226	AY.98.1
EPI_ISL_3824227	AZ.2
EPI_ISL_3824228	AY.43
EPI_ISL_3824229	AY.43
EPI_ISL_3824230	AY.102
EPI_ISL_3824270	AY.12
EPI_ISL_4251188	AY.43
EPI_ISL_4251192	AY.43
EPI_ISL_4251219	AY.43
EPI_ISL_4392868	AY.43
EPI_ISL_4395046	AY.9.1
EPI_ISL_4395061	AY.43
EPI_ISL_4395072	AY.4
EPI_ISL_4395085	AY.104
EPI_ISL_4395102	AY.86
EPI_ISL_4395109	AY.43
EPI_ISL_4395150	AY.33
EPI_ISL_4395151	AY.9.2
EPI_ISL_4395152	AY.102
EPI_ISL_4395153	AY.9.2
EPI_ISL_4395154	AY.43
EPI_ISL_4395155	AY.42
EPI_ISL_4395156	AY.98.1
EPI_ISL_4395157	AY.4
EPI_ISL_4395158	AY.43
EPI_ISL_4395159	AY.4
EPI_ISL_4395160	B.1.617.2
EPI_ISL_4395161	B.1.617.2
EPI_ISL_4395162	B.1.617.2

EPI_ISL_4395163	AY.9.2
EPI_ISL_4395165	AY.9.2
EPI_ISL_4395166	AY.43
EPI_ISL_4395167	AY.9.2
EPI_ISL_4395168	AY.43
EPI_ISL_4395169	AY.46
EPI_ISL_4395170	AY.9.2
EPI_ISL_4395173	AY.9.2
EPI_ISL_4395288	AY.5
EPI_ISL_4395290	AY.5.4
EPI_ISL_4395301	AY.73
EPI_ISL_4395303	AY.43
EPI_ISL_4395308	AY.25
EPI_ISL_4410796	AY.43
EPI_ISL_4410804	AY.9.2
EPI_ISL_4410806	AY.4
EPI_ISL_4410862	AY.42
EPI_ISL_4410869	AY.46.6
EPI_ISL_4410890	AY.4
EPI_ISL_4450796	AY.102
EPI_ISL_4450798	B.1.617.2
EPI_ISL_4450800	AY.43
EPI_ISL_4450829	AY.42
EPI_ISL_4450830	AY.9.2
EPI_ISL_4450831	AY.9.2
EPI_ISL_4450832	B.1.617.2
EPI_ISL_4450833	AY.4
EPI_ISL_4450834	AY.4
EPI_ISL_4450835	AY.43
EPI_ISL_4450836	AY.98.1
EPI_ISL_4450837	AY.46.6
EPI_ISL_4450838	AY.46.6

EPI_ISL_4450839	AY.43
EPI_ISL_4450840	AY.9.2
EPI_ISL_4450841	AY.98.1
EPI_ISL_4450888	AY.42
EPI_ISL_4450889	AY.43
EPI_ISL_4450892	AY.33
EPI_ISL_4450897	AY.43
EPI_ISL_4450901	AY.43
EPI_ISL_4450904	AY.43
EPI_ISL_4542104	AY.25
EPI_ISL_4542107	AY.39
EPI_ISL_4542117	AY.23
EPI_ISL_4542185	AY.43
EPI_ISL_4571409	B.1.617.2
EPI_ISL_4571425	AY.43
EPI_ISL_4814044	B.1.617.2
EPI_ISL_4814050	AY.102
EPI_ISL_4814097	AY.43
EPI_ISL_4814115	AY.43
EPI_ISL_4903661	AY.102
EPI_ISL_4903668	AY.43
EPI_ISL_5307621	B.1.617.2
EPI_ISL_5307655	AY.43
EPI_ISL_5307682	B.1.617.2
EPI_ISL_5307686	AY.4.3
EPI_ISL_5307719	AY.43
EPI_ISL_5307727	AY.111
EPI_ISL_5307728	AY.46
EPI_ISL_5307730	AY.43
EPI_ISL_5509155	AY.43
EPI_ISL_5766178	AY.44
EPI_ISL_6307802	AY.4

EPI_ISL_6307803	B.1.617.2
EPI_ISL_6307804	AY.25
EPI_ISL_6307805	AY.102
EPI_ISL_6307806	AY.102
EPI_ISL_6307807	AY.43
EPI_ISL_6307808	AY.4
EPI_ISL_6307809	AY.42
EPI_ISL_6307810	AY.43
EPI_ISL_6307811	AY.25
EPI_ISL_6307812	AY.102
EPI_ISL_6307813	AY.102

Table S3. Baseline characteristics for all individuals in the database with a confirmed breakthrough infection.

Variable	Level	Overall	% missing
n		488	
Gender (%)	Male<br>Female	231 (47.3%)<br>257 (52.7%)	0
Age, median (IQR) in years		44.5 (32.00-64.00)	0
Vaccine type, n (%)	Moderna<br>Pfizer/BioNTech	257 (52.2%)<br>231 (47.0%)	0
Time full vaccine to 1st symptom, median (IQR)		78 (47-122)	7.6
Time full vaccine/Delta outbreak to 1st symptoms, median (IQR)		50 (29-72)	49.0
Time full vaccine to 1st symptoms for Alpha & Delta patients vaccinated after Delta outbreak, median (IQR)		42 (24.75-58.75)	84
Time full vaccine to positive test, median (IQR)		78 (47-123.5)	0.2
Time full vaccine/Delta outbreak to positive test, median (IQR)		50 (30-72)	45.3
Time full vaccine to positive test for Alpha & Delta patients vaccinated after Delta outbreak, median (IQR)		45 (25-60)	82.2
Indication for vaccine			
Chronic diseases, n (%)	No<br>Yes	332 (68%)<br>156 (32%)	0

Healthcare worker, n (%)	No<br>Yes	429 (87.9%)<br>59 (12.1%)	0
Close contact to high-risk person, n (%)	No<br>Yes	363 (74.4%)<br>125 (25.6%)	0
Serious side effects to other vaccines, n (%)	No<br>Yes	487 (99.8%)<br>1 (0.2%)	0
Severe immunosuppression, n (%)	No<br>Yes	463 (94.9%)<br>25 (5.1%)	0
Acute fever, n (%)	No<br>Yes	488 (100%)<br>0 (0%)	0
Recovered from COVID-19 (%)	No<br>Yes	482 (98.8%)<br>6 (1.2%)	0
Pregnant (%)	No<br>Yes	487 (99.8%)<br>1 (0.2%)	0
PCR based Ct value, median (IQR)		20.45 (17.80 - 26.23)	60.2
Clinical severity (%)	Asymptomatic<br>Mild illness<br>Moderate illness<br>Severe illness<br>Critical illness and death	26 (5.3%)<br>448 (91.8%)<br>7 (1.4%)<br>5 (1.0%)<br>2 (0.4%)	0
Hospitalisation (%)	No<br>Yes	474 (97.1%)<br>14 (2.9%)	0
Suspected source of infection (%)	Club, party, bar<br>Cultural event<br>Family<br>Friends<br>Holidays<br>Hospital<br>Religion<br>Retirement and nursing home<br>School<br>Sports<br>Unknown<br>Work	17 (3.5%)<br>7 (1.4%)<br>134 (21.5%)<br>25 (5.1%)<br>105 (21.5%)<br>7 (1.4%)<br>3 (0.6%)<br>23 (4.7%)<br>9 (1.8%)<br>4 (0.8%)<br>118 (24.2%)<br>36 (7.4%)	0
Baseline risk factor			
Chronic lung disease (%)	No<br>Yes	454 (93%)<br>34 (7%)	0

Chronic renal disease (%)		No<br>Yes	480 (98.4%)<br>8 (1.6%)	0
Cancer (%)		No<br>Yes	473 (96.9%)<br>15 (3.1%)	0
Cardiovascular disease (%)		No<br>Yes	448 (91.8%)<br>40 (8.2%)	0
Arterial disease (%)		No<br>Yes	413 (84.8%)<br>74 (15.2%)	0.2
Diabetes (%)		No<br>Yes	447 (91.6%)<br>41 (8.4%)	0
Obesity (%)		No<br>Yes	485 (99.4%)<br>3 (0.6%)	0

Table S4. Baseline characteristics of patients with breakthrough infection who showed asymptomatic or mild illness (n=478) compared to patients with moderate, severe and critical illness (n=478).

**Variable**	**Level**	**asympto**	**mild**	**moderate**	**severe**	**critical/ death**	**% mis s**
n		26	448	7	5	2	
Age, med (IQR)	Years	67 (32.5-82.25)	43 (42-62)	77 (63-81)	81 (79-87)	87 (85-89)	0
Sex, n (%)	Man<br>Woman	10 (38.5%)<br>16 (61.5%)	212 (47.3%)<br>236 (52.7%)	5 (71.4%)<br>2 (28.6%)	3 (60%)<br>2 (40%)	1 (50%)<br>1 (50%)	0
Type of vaccine, n (%)	Moderna<br>Pfizer/BioNTech	13 (50%)<br>13 (50%)	239 (53.3%)<br>209 (46.7%)	4 (57.1%)<br>3 (42.9%)	1 (20%)<br>4 (80%)	0 (0%)<br>2 (100%)	0
Variants	Alpha<br>Delta	2 (25%)<br>6 (75%)	7 (2.8%)<br>242 (97.2%)	0 (0%)<br>5 (100%)	2 (50%)<br>2 (50%)	1 (50%)<br>1 (50%)	45.1
Indication for vaccination							
Chronic diseases, n (%)	No<br>Yes	13 (50%)<br>13 (50%)	318 (71%)<br>130 (29%)	0 (0%)<br>7 (100%)	1 (20%)<br>4 (80%)	0 (0%)<br>2 (100%)	0
Healthcare worker, n (%)	No<br>Yes	23 (88.5%)<br>3 (11.5%)	392 (87.5%)<br>56 (12.5%)	7 (100%)<br>0 (0%)	5 (100%)<br>0 (0%)	2 (100%)<br>0 (0%)	0

Close contact to high-risk person, n (%)	No<br>Yes	14 (53.8%)<br>12 (46.2%)	341 (76.1%)<br>107 (23.9%)	5 (71.4%)<br>2 (28.6%)	3 (60%)<br>2 (40%)	0 (0%)<br>2 (100%)	0
Serious side effects to other vaccines, n (%)	No<br>Yes	26 (100%)<br>0 (0%)	447 (99.8%)<br>1 (0.2%)	7 (100%)<br>0 (0%)	5 (100%)<br>0 (0%)	2 (100%)<br>0 (0%)	0
Severe immunosup pression, n (%)	No<br>Yes	24 (92.3%)<br>2 (7.7%)	428 (95.5%)<br>20 (4.5%)	5 (71.4%)<br>2 (28.6%)	4 (80%)<br>1 (20%)	2 (100%)<br>0 (0%)	0
Acute fever, n (%)	No<br>Yes	26 (100%)<br>0 (0%)	448 (100%)<br>0 (0%)	7 (100%)<br>0 (0%)	5 (100%)<br>0 (0%)	2 (100%)<br>0 (0%)	0
Pregnant, n (%)	No<br>Yes	26 (100%)<br>0 (0%)	447 (99.8%)<br>1 (0.2%)	7 (100%)<br>0 (0%)	5 (100%)<br>0 (0%)	2 (100%)<br>0 (0%)	0
Recovered from COVID19, n (%)	No<br>Yes	25 (96.2%)<br>1 (3.8%)	443 (98.9%)<br>5 (1.1%)	7 (100%)<br>0 (0%)	5 (100%)<br>0 (0%)	2 (100%)<br>0 (0%)	0
Time vacc to 1st symptoms, median (IQR)	days	148 (148-148)	77 (47-119)	191.5 (109.3-244.5)	123 (104-167.5)	122 (122-122)	7.6
Time vacc/Delta outbreak to 1st symptoms, median (IQR)	days	107 (107-107)	50 (29-70)	106.5 (98.3-108.3)	64 (63-65)	25 (25-25)	49
Time vacc to 1st symptoms for Alpha & Delta patients vaccinated after Delta outbreak,	days	NA	42 (24.75-58.75)	NA	NA	NA	84

median (IQR)							
Time vacc to positive test, median (IQR)	days	71.5 (41.25-135.25)	78 (48-120.5)	150 (96.5-242)	85 (2-123)	62 (32-92)	0.2
Time vacc/Delta outbreak to positive test, median (IQR)	days	36 (20.75-57.5)	50 (31.75-72)	107 (78-109)	32 (2-63.75)	13.5 (7.75-19.25)	45.3
Time vacc to positive test for Alpha & Delta patients vaccinated after Delta outbreak, median (IQR)	days	66.5 (44.25-88.75)	45.5 (27.25-60.5)	NA	2 (2-2)	2 (2-2)	82.2
PCR ct value, median (IQR)		28.8 (28.35-31.31)	20.27 (17.67-25.7)	25.03 (23.38-26.68)	NA	23.21 (23.21-23.21)	60.2
Hospitalisation	No<br>Yes	24 (92.3%)<br>2 (7.7%)	447 (99.8%)<br>1 (0.2%)	1 (14.3%)<br>6 (85.7%)	2 (40%)<br>3 (60%)	0 (0%)<br>2 (100%)	0
Source of infection	Club, party, bar<br>Cultural event<br>Family<br>Friends<br>Holidays<br>Hospital<br>Religion<br>Retirement and nursing home<br>School<br>Sports<br>Unknown<br>Work	1 (3.8%)<br>0 (0%)<br>5 (19.2%)<br>1 (3.8%)<br>4 (15.4%)<br>0 (0%)<br>0 (0%)<br>8 (30.8%)<br>0 (0%)<br>0 (0%)<br>7 (26.9%)<br>0 (0%)	16 (3.6%)<br>7 (1.6%)<br>125 (27.9%)<br>24 (5.4%)<br>101 (22.5%)<br>7 (1.6%)<br>3 (0.7%)<br>11 (2.5%)<br>9 (2%)<br>4 (0.9%)<br>105 (23.4%)<br>36 (8%)	0 (0%)<br>0 (0%)<br>4 (57.1%)<br>0 (0%)<br>0 (0%)<br>0 (0%)<br>0 (0%)<br>1 (14.3%)<br>0 (0%)<br>0 (0%)<br>2 (28.6%)<br>0 (0%)	0 (0%)<br>0 (0%)<br>0 (0%)<br>0 (0%)<br>0 (0%)<br>0 (0%)<br>0 (0%)<br>2 (40%)<br>0 (0%)<br>0 (0%)<br>3 (60%)<br>0 (0%)	0 (0%)<br>0 (0%)<br>0 (0%)<br>0 (0%)<br>0 (0%)<br>0 (0%)<br>0 (0%)<br>1 (50%)<br>0 (0%)<br>0 (0%)<br>1 (50%)<br>0 (0%)	0
Baseline risk factor							

Chronic lung disease	No<br>Yes	20 (76.9%)<br>6 (23.1%)	422 (94.2%)<br>26 (5.8%)	6 (85.7%)<br>1 (14.3%)	4 (80%)<br>1 (20%)	2 (100%)<br>0 (0%)	0
Chronic renal disease	No<br>Yes	26 (100%)<br>0 (0%)	443 (98.9%)<br>5 (1.1%)	5 (71.4%)<br>2 (28.6%)	5 (100%)<br>0 (0%)	1 (50%)<br>1 (50%)	0
Cancer	No<br>Yes	25 (96.2%)<br>1 (3.8%)	436 (97.3%)<br>12 (2.7%)	6 (85.7%)<br>1 (14.3%)	4 (80%)<br>1 (20%)	2 (100%)<br>0 (0%)	0
Cardiac disease	No<br>Yes	23 (88.5%)<br>3 (11.5%)	422 (94.2%)<br>26 (5.8%)	2 (28.6%)<br>5 (71.4%)	1 (20%)<br>4 (80%)	0 (0%)<br>2 (100%)	0
Arterial hypertensio n	No<br>Yes	22 (84.6%)<br>4 (15.4%)	384 (85.9%)<br>63 (14.1%)	4 (57.1%)<br>3 (42.9%)	3 (60%)<br>2 (40%)	0 (0%)<br>2 (100%)	0
Diabetes	No<br>Yes	25 (96.2%)<br>1 (3.8%)	422 (92%)<br>36 (8%)	4 (57.1%)<br>3 (42.9%)	4 (80%)<br>1 (20%)	2 (100%)<br>0 (0%)	0
Obesity	No<br>Yes	26 (100%)<br>0 (0%)	446 (99.6%)<br>2 (0.4%)	6 (85.7%)<br>1 (14.3%)	5 (100%)<br>0 (0%)	2 (100%)<br>0 (0%)	0

Table S5. Chronic diseases and conditions leading to an especially high risk for severe course of disease according to the Federal Office of Public Health. According to the FOPH, only adults are considered to be at high risk. Therefore, the following criteria refer only to adults.

Condition	Specific disease	Details
Adults with Trisomy 21		
Hypertension	Arterial hypertension with end-organ damage	
	Therapy-resistant arterial hypertension	
Cardiovascular disease	General	NYHA II-IV and NT-proBNP > 125 pg/ml
		2 cardio-vascular risk factors
		Prior stroke and/or symptomatic vasculopathy
		Chronic renal insufficiency (Stage 3, GFR <60 ml/min)
	Coronary heart disease	Myocardial infarction in the past 12 months
		Symptomatic chronic coronary syndrome despite medical treatment

	Disease of the heart valves	Moderate or serious stenosis and/or regurgitation in addition to meeting at least one general criterion
		Any surgical or percutaneous valve replacement in addition to meeting at least one general criterion
	Cardiac insufficiency	Patients with dyspnea of functional class NYHA II–IV or NT-proBNP > 125pg/ml despite medical treatment for any LVEF (HFpEF, HFmrEF, HFrEF)
		Cardiomyopathy with any cause
		Pulmonary arterial hypertension
	Arrhythmia	Auricular fibrillation with a CHA2DS2-VASc score of at least 2 points
		Prior implant of pacemaker (incl. ICD and/or CRT implantation) in addition to meeting one general criterion
	Adults with congenital heart disease	Congenital heart disease according to the individual assessment of the attending cardiologist
Diabetes	Diabetes mellitus, with long-term complications or a HbA1c of ≥ 8%	
Chronic pulmonary and respiratory disease	Chronic obstructive lung diseases GOLD Grade II-IV	
	Pulmonary emphysema	
	Uncontrolled asthma, in particular serious bronchial asthma	
	Interstitial lung diseases / pulmonary fibrosis	
	Active lung cancer	
	Pulmonary arterial hypertension	
	Pulmonary vascular disease	
	Active Sarcoidosis	
	Cystic fibrosis	
	Chronic lung infections (atypical mycobacteriosis, bronchiectasis, etc.)	

	Ventilated patients	
	Diseases with severely reduced lung capacity	
Diseases/Therapies that weaken the immune system	Serious immunosuppression (e.g. HIV-infection with a CD4+ T cell number of < 200µl)	
	Neutropenia (<1'000 neutrophils/µl) for ≥1 week	
	Lymphocytopenia (<200 lymphocytes/µl)	
	Hereditary immunodeficiencies	
	Use of medication that suppresses the immune defenses (such as long-term use of glucocorticoids (prednisolone equivalent > 20 mg/day), monoclonal antibodies, cytostatics, biologics etc.)	
	Aggressive lymphomas (all entities)	
	Acute lymphatic leukaemia	
	Acute myeloid leukaemia	
	Acute promyelocytic leukaemia	
	T-cell prolymphocytic leukaemia	
	Primary lymphomas of the central nervous system	
	Stem cell transplantation	
	Amyloidosis (light-chain (AL) amyloidosis)	
	Chronic lymphatic leukaemia	
	Multiple myeloma	
	Sickle-cell disease	
	Bone marrow transplant	
	Solid organ transplant	
	Individuals on a transplant waiting list	
Cancer	Cancer undergoing medical treatment	

Obesity	Patients with a body-mass index (BMI) of 35 kg/m2 or more
Liver disease	Cirrhosis of the liver
Renal disease	Chronic renal insufficiency with a GFR < 60 ml/min

Table S6. Multivariable logistic regression exploring the relationships between potential risk factors and the probability to suffer from a SARS-CoV-2 breakthrough infection using the ’control’ group 2. Confidence intervals and p-values are computed using Firth penalization, because the level “Yes'' for “Acute fever” was rarely observed. Only Moderna and Pfizer/BioNTech vaccines are compared, because no other vaccine was used in the control population.

	**n**	**Odds ratio**	**CI**	**p**
Intercept	107039	0.010	(0.008-0.013)	<0.001
Age		0.982	(0.977-0.987)	<0.001
Gender (woman vs. man)		1.048	(0.876-1.255)	0.606
Vaccine (Pfizer/BioNTech, ref =Moderna		1.530	(1.342-1.743)	<0.001
Chronic disease (=Yes)		1.990	(1.590-2.482)	<0.001
Healthcare worker (=Yes)		1.927	(1.438-2.538)	<0.001
Close contact with high-risk person (=Yes)		0.934	(0.752-1.153)	0.530
Pregnant (=Yes)		1.265	(0.144-4.581)	0.784
Severe immunosuppression (=Yes)		1.248	(0.806-1.849)	0.307
Serious side-effects from previous vaccines (=Yes)		0.289	(0.033-1.035)	0.058
Acute fever (=Yes)		2.693	(0.021-19.002)	0.549
Recovered from COVID-19 (=Yes)		0.431	(0.178-0.861)	0.014

Table S7. Multivariable logistic regression exploring the relationships between potential risk factors and the probability to suffer from a Delta variant caused breakthrough infection using the ’control’ set 1 and 2. Only Moderna and Pfizer/BioNTech vaccines are compared, because no other vaccine was used in the control populations.

	**Control set 1**				**Control set 2**			
	**n**	**Odds ratio**	**CI**	**p**	**n**	Odds ratio	CI	p
Intercept	125076	0.004	(0.003-0.006)	<0.001	106807	0.006	(0.004-0.009)	<0.001
Age		0.985	(0.978-0.991)	<0.001		0.979	(0.972-0.986)	<0.001
Gender (woman vs. man)		0.998	(0.779-1.280)	0.988		1.008	(0.787-1.293)	0.947

Vaccine (Pfizer/BioNTech, ref =Moderna	1.473	(1.232-1.759)	<0.001	1.551	(1.296-1.856)	<0.001
Chronic disease (=Yes)	1.972	(1.444-2.674)	<0.001	1.885	(1.374-2.568)	<0.001
Healthcare worker (=Yes)	1.576	(1.061-2.278)	0.019	2.184	(1.484-3.126)	<0.001
Close contact with high-risk person (=Yes)	0.918	(0.677-1.231)	0.575	0.957	(0.708-1.278)	0.768
Pregnant (=Yes)	1.247	(0.071-5.614)	0.826	2.331	(0.264-8.516)	0.367
Recovered from COVID19 (=Yes)	0.18	(0.030-0.561)	0.016	0.301	(0.063-0.851)	0.020

Table S8. Multivariable generalized linear model with binomial distribution investigating the probability of suffering from a breakthrough infection caused by the Delta variant (as compared to the Alpha variant taken as the reference) as a function of individual characteristics. Confidence intervals and p-values are computed using Firth penalization due to rare or no observations in some factors' levels.

	n	Odds ratio	CI	p
Intercept	268	8507.26	(199.79, 8455840.37)	<0.001
Age		0.93	(0.85-0.98)	0.007
Gender (woman vs. man)		1.05	(0.21-5.39)	0.951
Vaccine Pfizer/BioNTech, (ref=Moderna)		0.24	(0.03-0.90)	0.033
Healthcare worker (=Yes)		3.55	(0.11-843.96)	0.516
Close contact with high-risk person (=Yes)		0.62	(0.13-2.90)	0.534
Severe immunosuppression (=Yes)		0.79	0.05-14.70	0.868
Pregnant (=Yes)		0.01	(0.00-4.15)	0.116
Chronic disease (=Yes)		0.12	0.01-1.20	0.071
Chronic lung disease (=Yes)		1.37	0.18-22.72	0.780
Chronic renal disease (=Yes)		0.48	0.02-12.17	0.641
Cancer (=Yes)		0.33	0.01-5.07	0.430
Cardiac disease (=Yes)		0.69	0.10-5.12	0.706
Arterial hypertension (=Yes)		6.93	1.26-57.33	0.025
Diabetes (=Yes)		3.09	0.43-35.82	0.278
Obesity (=Yes)		0.05	(0.00-1.16)	0.061

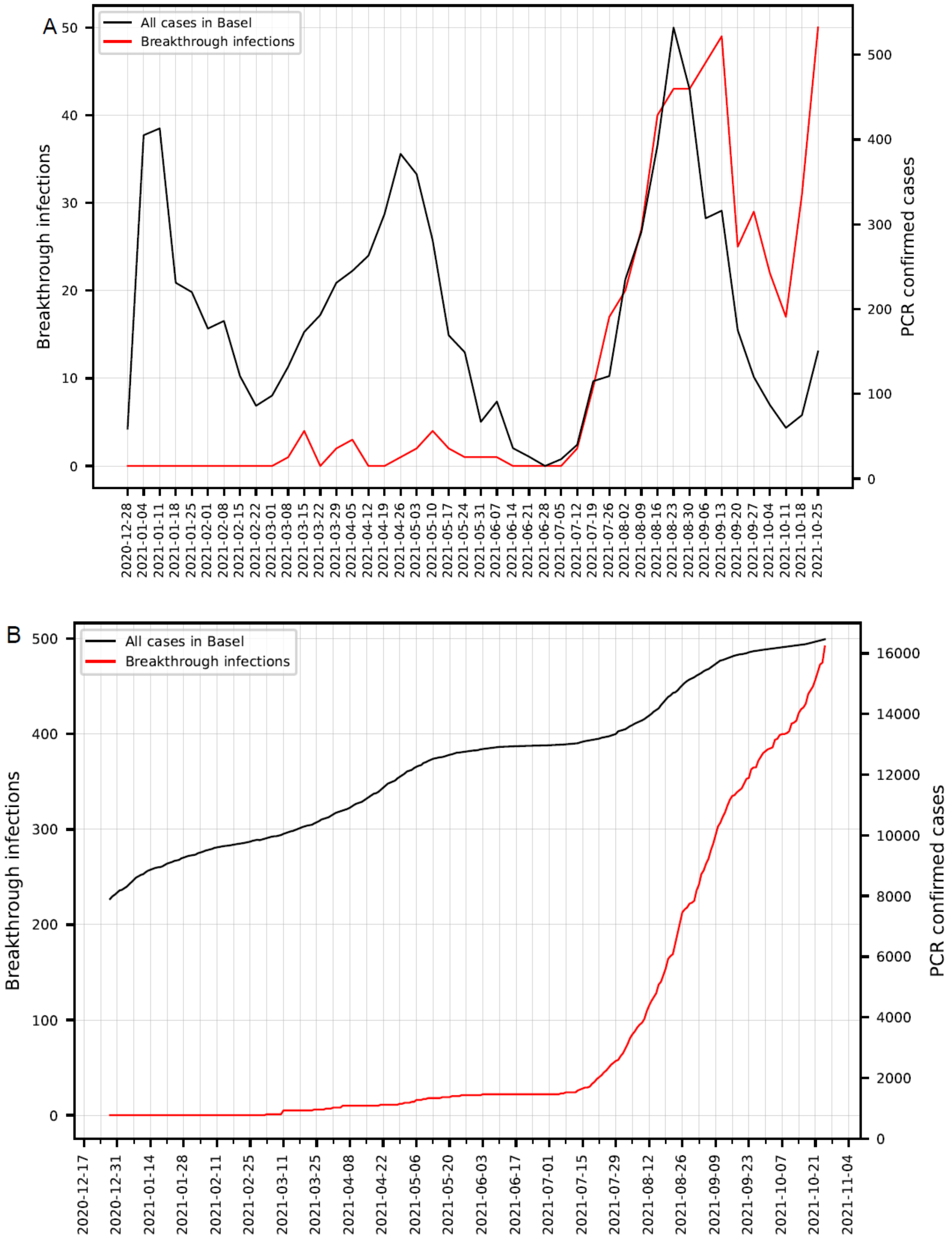

Figure S1. SARS-CoV-2 PCR confirmed cases after introduction of the vaccine. The red line shows all breakthrough infections and the black line shows all PCR confirmed cases (secondary axis) in Basel City

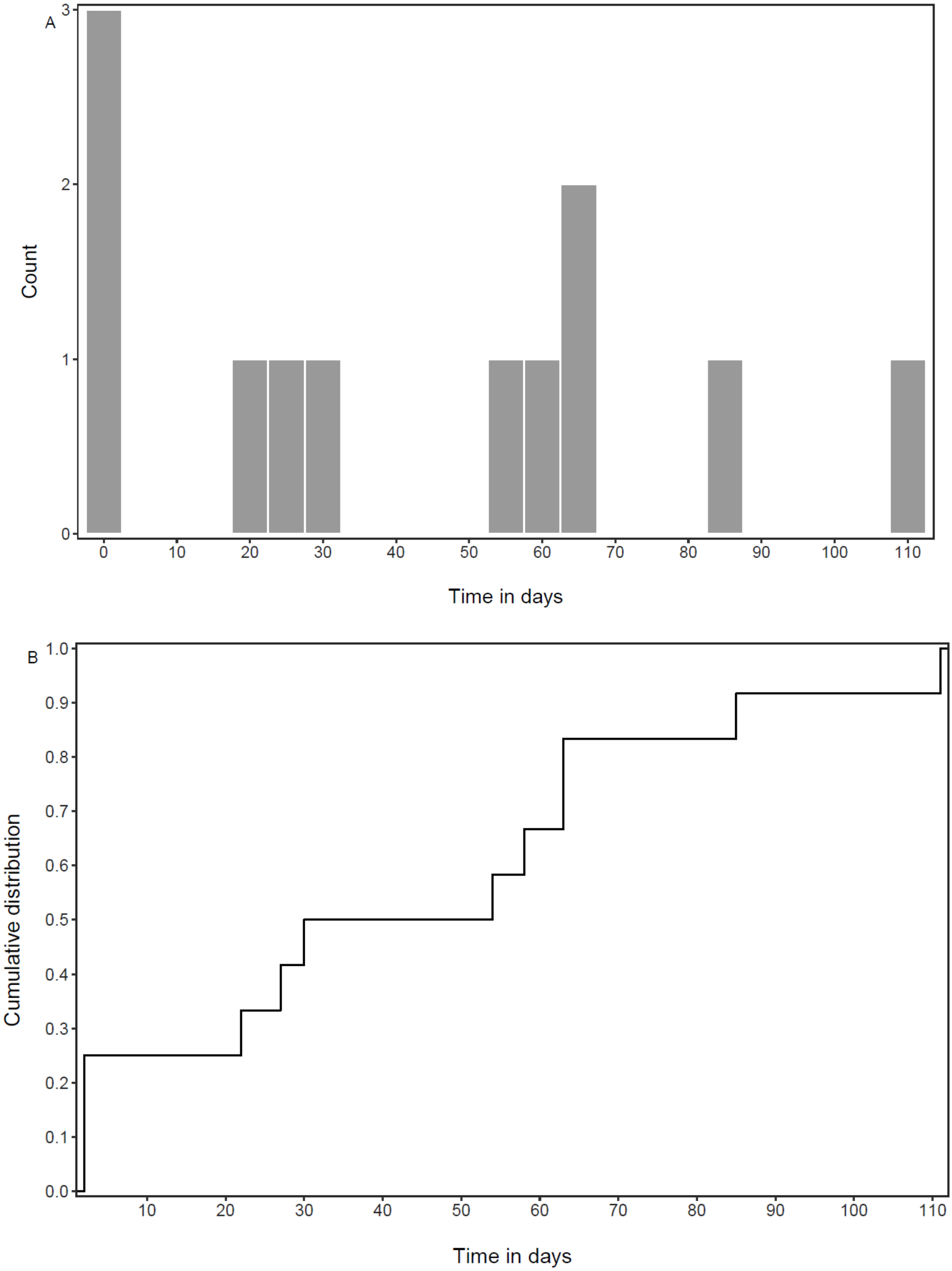

Figure S2. Distribution time in days from full vaccination (as defined) to the breakthrough infection with the Alpha variant. (A), absolute frequency; (B) cumulative frequency.

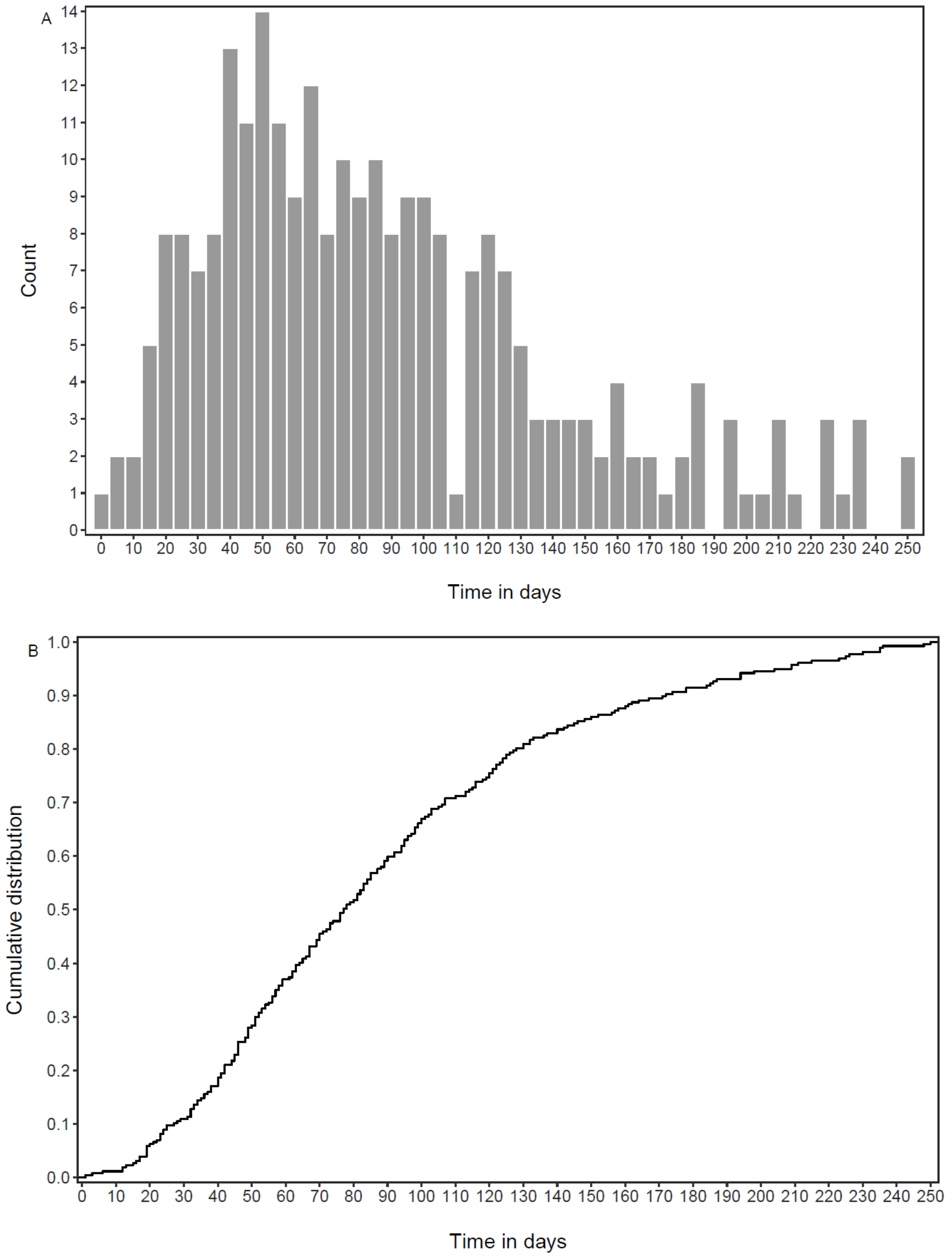

Figure S3. Distribution time in days from full vaccination (as defined) to the breakthrough infection with the Delta variant. (A), absolute frequency; (B) cumulative frequency.

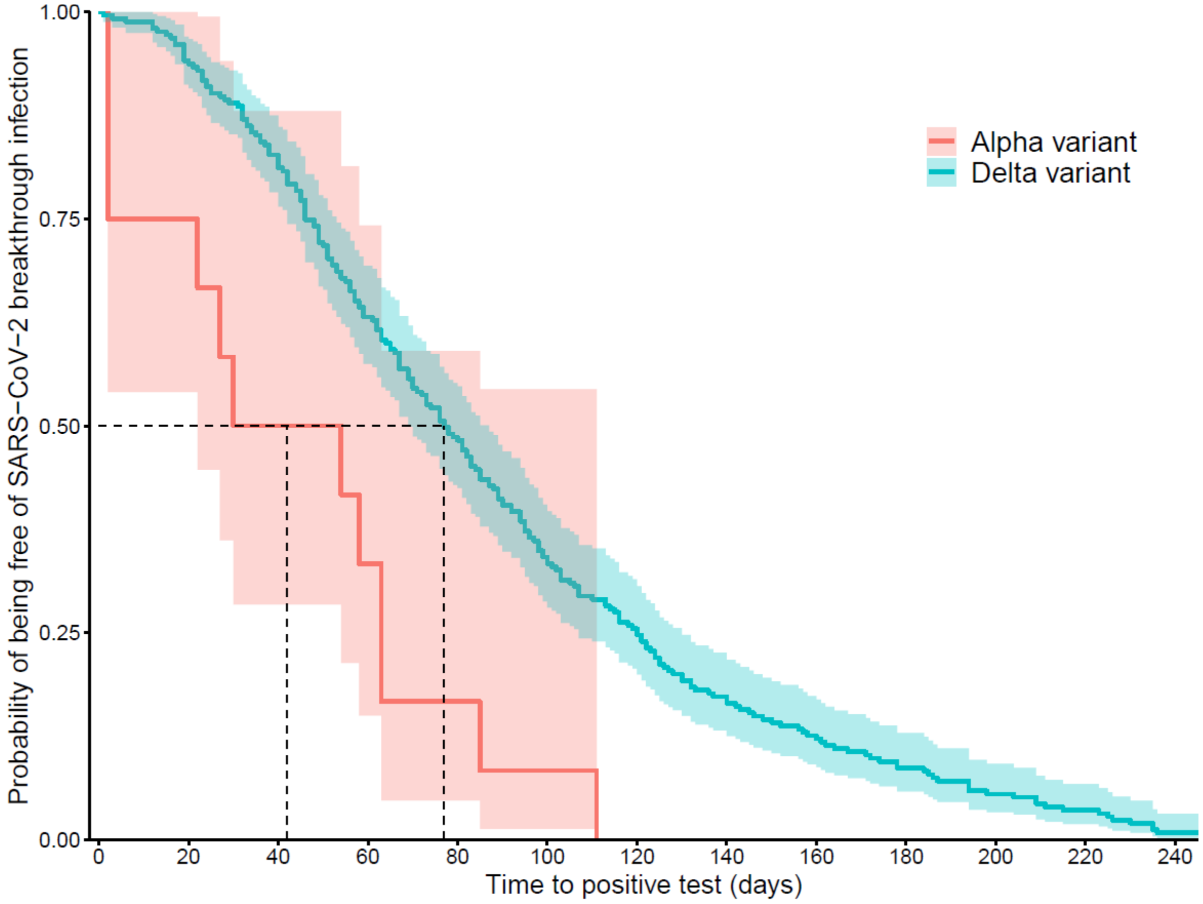

Figure S4. Kaplan-Meier curve representing the proportion of patients free of breakthrough infection in relation to the time elapsed between the vaccination and the positive test for the Alpha and the Delta variant.
